# Decomposition of cell activities revealing the role of the cell cycle in driving biofunctional heterogeneity

**DOI:** 10.1038/s41598-021-02926-4

**Published:** 2021-12-06

**Authors:** Tian Lan, Meng Yu, Weisheng Chen, Jun Yin, Hsiang-Tsun Chang, Shan Tang, Ye Zhao, Spyros Svoronos, Samuel W. K. Wong, Yiider Tseng

**Affiliations:** 1 Innovation Research Institute of Traditional Chinese Medicine, Shandong University of Traditional Chinese Medicine, Jinan, 250355 Shandong China; 2 Department of Chemical Engineering, University of Florida, Gainesville, FL 32611 USA; 3 Department of Statistics and Actuarial Science, University of Waterloo, Waterloo, ON N2L 3G1 Canada

**Keywords:** Cell adhesion, Cell migration, Cellular imaging, Cytoskeleton, Data acquisition, Data processing, Functional clustering, Image processing, Biophysics, Biotechnology, Cell biology, Computational biology and bioinformatics

## Abstract

Heterogeneity of cell phenotypes remains a barrier in progressing cell research and a challenge in conquering cancer-related drug resistance. Cell morphology, the most direct property of cell phenotype, evolves along the progression of the cell cycle; meanwhile, cell motility, the dynamic property of cell phenotype, also alters over the cell cycle. However, a quantifiable research understanding the relationship between the cell cycle and cell migration is missing. Herein, we coordinate the migratory behaviours of NIH 3T3 fibroblasts to their corresponding phases of the cell cycle, the G1, the S, and the G2 phases, and explain the relationship through the spatiotemporal arrangements between the Rho GTPases’ signals and cyclin-dependent kinase inhibitors, p21^Cip1^, and p27^Kip1^. Taken together, we demonstrate that both cell morphology and the dynamic subcellular behaviour are homogenous within each stage of the cell cycle phases but heterogenous between phases through quantitative cell analyses and an interactive molecular mechanism between the cell cycle and cell migration, posing potential implications in countering drug resistance.

## Introduction

Both cell migration and proliferation play essential roles in physiological and pathological events, including embryo development, wound repairs, and cancer metastasis. During vertebrate morphogenesis, cell migration tightly associated in vascular sprout and development of epithelial sheets or clusters^1–3^. In cancer metastasis, cells grow, extravasate, and eventually migrate to remote tissues, where they proliferate again to establish secondary tumors^4^. Although proliferation and migration are two pertinent cellular activities that commonly coexist, the interactive molecular mechanism(s) connecting these two cell activities is still poorly understood.

Cell proliferation is funneled by the cell cycle, which is regulated by the cyclin-dependent kinases (CDKs)^5–7^. When CDKs are activated, they promote the progression of cell cycle as well as the alternation of gene transcription pattern that affects cell behaviour. For example, when CDK4 and CDK6 trigger the phase transition from the G1 phase of the cell cycle to the S phase, they phosphorylate and inactivate the retinoblastoma protein Rb, leading to the expression of a subset of proliferation-associated E2F target genes^8,9^. Hence, it is widely accepted that the alternation of cell morphologies and dynamics is a natural consequence of the cell cycle progression on a more rudimentary level. Despite observations surrounding these processes, the cell cycle’s impact on cell dynamics has not been systematically addressed before. Cell migration requires cytoskeletal remodeling, for which Rho GTPases serve as the primary molecular switches^10,11^. When RhoA, one of the Rho GTPases, is activated, it propagates its signal to downstream proteins, Rho Kinase (ROCK), then to LIM kinase (LIMK) to inhibit ADF/cofilin in its F-actin severing and G-actin sequestering activities^12^. The crosstalk between the CDKs and RhoA pathways that relates the cell cycle and cell migration have only been mentioned through p27^Kip1^ and p21^Cip1^, two CDK inhibitors (CDKIs)^13–16^.

As the cell is quiescent in the G1 phase, p27^Kip1^ and p21^Cip1^ tightly bind to the cyclin-CDK complexes in the nucleus to prevent the cells from progressing in the cell cycle^17,18^. Upon mitogen stimulation, cells enter the S phase as these CDKIs dissociate from the cyclin-CDK complexes so that the chromosome can be duplicated. Consequently, p27^Kip1^ is exported from the nucleus to the cytoplasmic region to bind and inhibit RhoA before the p27^Kip1^-RhoA complex is degraded^13,14^. Meanwhile, p21^Cip1^ binds to and inhibits the ROCK^16^. Since the RhoA signaling is disrupted by these two CDKIs after the G1/S phase transition, the reactivation of ADF/cofilin should allow actin-cytoskeletal remodeling^12,19,20^. As a result, it has been speculated that CDKIs play a dual role in tumorigenesis to release corresponding cells from cell cycle arrest and in metastasis to enhance actin-cytoskeletal dynamics^14,15,21–23^.

This perspective suggests that the interactions among CDKIs and proteins in the RhoA pathway after the G1/S phase transition increase the likelihood for the metastasis of cancer cells whereas cell spread events likely occur in the G2 phase. However, previous studies have reported that cell motility in the G1/S phases is greater than in the G2/M phases for all the cell types that have been surveyed, including L929, HeLa, and BT4Cn glioma cells^24^. Additionally, NBT-II rat bladder carcinoma cells respond to fibroblast growth factor-1 and increase their dynamics only in the G1/S phases but not in the G2 phase^25^. These cases reveal that the surveyed cancer cells only have their motility reduced after passing the G1/S phase transition and are against the proposal for the roles of these two CDKIs in cancer metastasis. The gap in understanding whether cell motility should be used in evaluating cell invasion or whether we lack an integrative view for cell dynamics in the G2 cells makes it critical to provide a robust framework for the relationship between the cell cycle and cell migration.

Hence, in this study, we apply a robust cell migration assessment, the *CN correlation* analysis^26,27^ to probe the phenotype spectrum (*i.e.*, the alternation of cell morphologies and dynamics following the progression of cells through the stages of the cell cycle). The *CN correlation* analysis allows us to decipher the migratory phenotype of cells in each stages of the cell cycle effectively and to clarify the impact of the two aforementioned CDKIs on cell dynamics, hence obtaining the comprehensive relationship between cell migration and the cell cycle. Building this framework provides an architecture through which we can map the structural properties throughout the cell cycle and simplifies our understanding of cell physiology.

## Results

### The evolutions of both cell velocity and morphology stem from cell cycle progression

**Figure 1:**
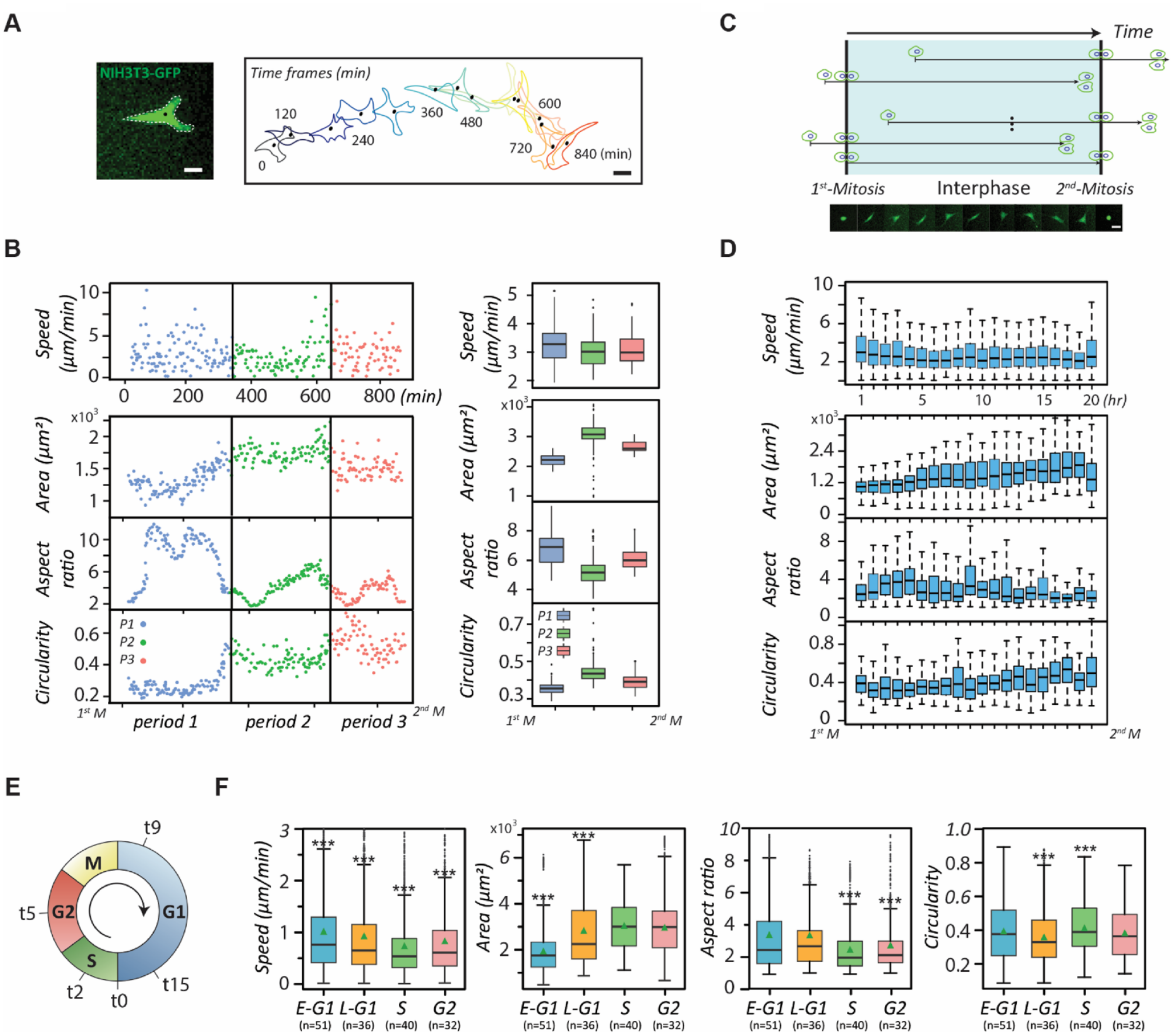
The evolution of migration and morphology of NIH fibroblasts over the cell cycle. (**A**) Green fluorescent protein (GFP)-labelled single NIH3T3 fibroblasts monitored at 3-min time intervals over an entire cell cycle beginning at cell-division. The time-evolved cell outlines and corresponding cell centroids over the entire cell cycle of a fibroblast are shown. Scale bar: 20 µm. (**B**) Scatter plots (left) and Box plots (right) showing the dynamic and morphological evolutions of single NIH 3T3 fibroblasts during the cell cycle progression. The cell phenotypes, including instantaneous speed, area, aspect ratio, and circularity were monitored at 3-min time intervals throughout the cell cycle. The perpendicular lines separate periods exhibiting different cell phenotypes. (**C**) The schematic of the time course alignments of live, single cells tracked over 20 h, related to Fig. 1D. The series of micrographs show the morphological evolution of the GFP-labeled NIH 3T3 fibroblasts over the whole cell cycle. Scale bar: 20 µm. (**D**) The box plots verifying cell phenotype evolutions over the cell cycle using 20 cells. (**E**) The plot representing the time of the optimal profiles of double-thymidine-synchronized cells that propagate to the early G1, the late G1, the S, and the G2 phase, determined as 9, 15, 2, and 5 h, respectively (and denoted as t9, t15, t2, and t5, respectively). (**F**) The box plots showing the dynamic and morphological measurements of synchronized NIH 3T3 fibroblasts (n > 20) in different stages of the cell cycle phases. Data information: *** indicates that the group of data is statistically significant compared to other groups. The statistical results of this figure are listed in Supplementary Table 1.

We first monitored whether NIH 3T3 fibroblasts possess significant phenotype transitions during the interphase of the cell cycle through a representative cell migratory trajectory and the corresponding cell shape (Fig. 1A). The results reveal that the trajectory is a straight line in the beginning but becomes curved later. Accordingly, the cell shape also transforms significantly. Thereafter, we scrutinized the motility and polarity of the cell at 3-min intervals over the whole cell cycle in terms of basic cell parameters, including instantaneous speed, area, aspect ratio, and circularity (Fig. 1B). The least-square criterion was also applied to classify the complete cell cycle into different periods based on the parameter similarities. The results suggest that the whole cell cycle could be divided into three distinct periods (see Materials and Methods).

The results show that the cell’s instantaneous speed fluctuates abruptly in the first and third periods but maintains consistently low in the second period. The cell area is initially small but continuously increases in the first period, steadies in the second period, and disperses vigorously in the third period. In terms of cell shape, the monitored cell displays a high aspect ratio and low circularity in the first period but contrasting results in the other periods, demonstrating that the cell shape transforms after the second period.

To verify that these results do not emerge from coincidence, we acquired 20 single-fibroblast movies, 20 h long each, ensuring inclusion of at least one cell division event. We then identified a movie containing a complete cell cycle with two consecutive cell divisions and set its timeframe as a benchmark to align the timeframes of the rest. If the cell division occurred at an earlier time in an aligned movie, then the division time of this movie would be superimposed with the first division of the standard one; otherwise, the division time would be overlaid with the second division of the standard one (Fig. 1C). Under these conditions, all cells possessed relatively similar timeframes with respect to the cell cycle. Hence, we could analyze cell phenotypes through the least-square criterion again using these four cell parameters over a reference timeframe by much larger sample sizes (Fig. 1D). We compared the individual trends of the mean values of those four parameters with their counterparts shown in Fig. 1B, left panel, the results suggest that those parameters in the cell cycle might exist the similar trends that can be distinguished into three periods with different performances.

The presence of three distinct phenotypes within the cell cycle seems to correlate with the progression of the cell cycle. Therefore, we further synchronized NIH 3T3 fibroblasts into different stages of the cell cycle (Fig. 1E and Supplementary Fig. 1) and randomly acquired 25 single-cell movies in each of these stages to see whether the phenotype transitions are coincident with the cell cycle progression (Fig. 1F). The results show that the instantaneous speeds are the highest in the G1 phase, reach a minimum in the S phase, and bounce back slightly in the G2 phase. The cell area displays a monotonically increasing trend until the late G1 phase and reaches a plateau after entering the S phase. The aspect ratio and circularity together suggest that cells have the lowest polarity in the S phase. In essence, cell phenotype transitions exhibit a strong correlation with the cell cycle phases, and these results lay the assumption that cell cycle stages could be the functional subpopulation of heterogeneity with phenotypic disparity.

### Overview: *CN-correlation* assessment

The above assessments have built a loose correlation between phenotype transitions and the cell cycle progression. However, traditional cell migration analyses prevent detailed investigation of cellular dynamic behaviours. Those techniques obligate a long period of cell trajectories (~ 10 h)^28,29^, which leads to a dilemma where the duration of the sampled cell trajectories must be long enough for statistically meaningful analysis, yet short enough to have the trajectories restrained within the same stage of the cell cycle phases. In addition, conventional assessments also fail to provide the underlying mechanisms explaining the causes of their results. These deficiencies led us to develop a novel biological model, termed the *CN correlation analysis*^26^, to statistically decode the cell dynamic process into contributing subcellular migratory activities by weights. This novel analysis only requires short time (~ one hour) cell movies as the raw data; hence, we can obtain the cell migratory details within a given stage of the cell cycle.

**Figure 2:**
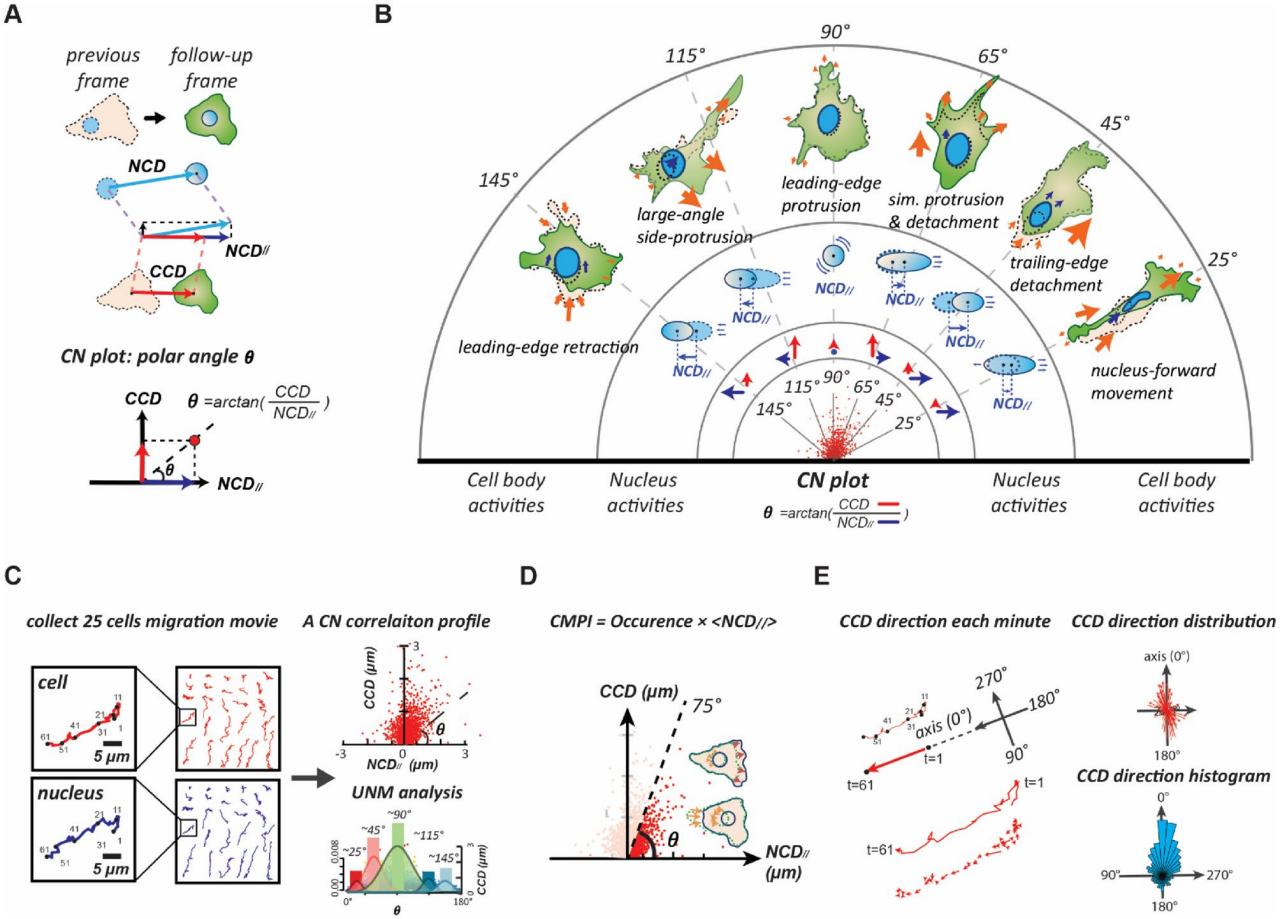
The overview of CN correlation analysis. (**A**) The framework of CN correlation analysis. (**B**) Six representative subcellular activities displaying their specific distributions in the CN plot. A signature CN polar angle represents a migratory pattern of the corresponding subcellular activity. (**C**) Illustration of the CN profile definition and the univariate normal mixtures (UNM) analysis. A CN correlation profile (n = 1500) is constructed using a collection of 25 randomly selected, one-hour movies of NIH fibroblasts at one-minute intervals. The UNM analysis is applied to profile the composition of normal distribution curves that describe the signature migration patterns and the proportions of the corresponding subcellular activities. (**D**) The schematic of the cell migration. Occurrence denotes the frequency rate of the CN correlations over populations located in the region (0°–75°); < NCD_//_ > represents the mean value of NCD_//_ over the population. (**E**) Methodology schematic for the migration polarity analysis of the cell. One-minute CCD direction is aligned with the orientation of one-hour displacement. The direction histogram reveals migratory polarity.

This method is described in brief: We identified each known cell locomotion event of the NIH 3T3 fibroblasts in single-cell movies that were recorded at one-minute intervals. Consequently, the cell centroid displacement between each pair of adjacent video frames of the cell locomotion movie was analyzed and denoted as a *CCD*, and the relative nuclear centroid displacement along the *CCD* direction was analyzed and denoted as the *NCD*_//_. Then, the pair of *CCD* and *NCD*_//_ determined a point in the *CCD vs. NCD*_//_ coordinate system (the *CN* plot), called the *CN correlation* datum (Fig. 2A). Through this procedure, we found that data generated by each specific locomotion event form a normal distribution pattern around a mean (peak) polar-angle in the *CN* plot. These cell locomotion are the nucleus-forward movement, which has the peak polar angle ~ 25°; the trailing-edge detachment, ~ 45°; the simultaneous protrusion and detachment, ~ 65°; the leading-edge protrusion, ~ 90°; the large-angle side-protrusion, ~ 115°; and the leading-edge retraction, ~ 145°. We denote these peak polar angles as signature polar angles for cell locomotion (Fig. 2B).

Following, the *CN correlation* analysis was applied to an undesignated cell movement event to reveal the percentages of individual cell locomotion contributing to the event. We recorded twenty-five randomly selected single NIH 3T3 fibroblast for one hour at one-minute intervals as individual cell movies to decipher the 1500 *CN correlation* data, which were plotted together to form the *CN* profile^26^. Then, the automatic univariate normal mixtures (*UNM*) algorithm was applied to the profile to statistically classify the histogram of the polar angles into appropriate normal distributions based on the signature polar angles to reveal the weight of each cell locomotion in the migratory event^27^ (Fig. 2C). Hence, the *CN* profile is a statistical descriptor that can analyze the unique cell migration pattern. This information can be utilized to further connect the underlying signaling pathways involved in each cell locomotion.

Cell motility is carried out by effective migration, in which a cell and its coupled nucleus must move in the same direction. In the *CN correlation* analysis, every datum with a low polar angle (*i.e.*, < 75°) possesses a nuclear displacement that effectively contributes to migration, while that with a high polar angle having an irrelevant *NCD*_//_. Hence, only the *CN correlation* data located within the polar angles ranging from 0° to 75° are considered to contribute to the effective migration and the values of all *NCD*_//_ with a low polar angle can be summed together to robustly estimate the cell motility of the probed cells, called the cell migration potential index (*CMPI*) (Fig. 2D). Our previous study^26^ has proved that a greater *CMPI* value indicates a greater long-term motility for a cell.

The *CN correlation* analysis possesses excellent applicability and reliability for cell migration assessment^26^. When we incorporated cell polarity into the *CN correlation* analysis, it can evaluate the persistence of mesenchymal cell migration. The consistency of the short-term *CCD* orientations concerning the long-term cell displacement indicates that the probed cell possesses high polarity (Fig. 2E). Hence, the *CN correlation* analysis can decompose the cellular phenotypes into functional subcellular events and accordingly assess cell migration from both the motility and polarity perspectives.

### Cell migration patterns are distinct and highly homogeneous in each stage of the cell cycle phase

**Figure 3:**
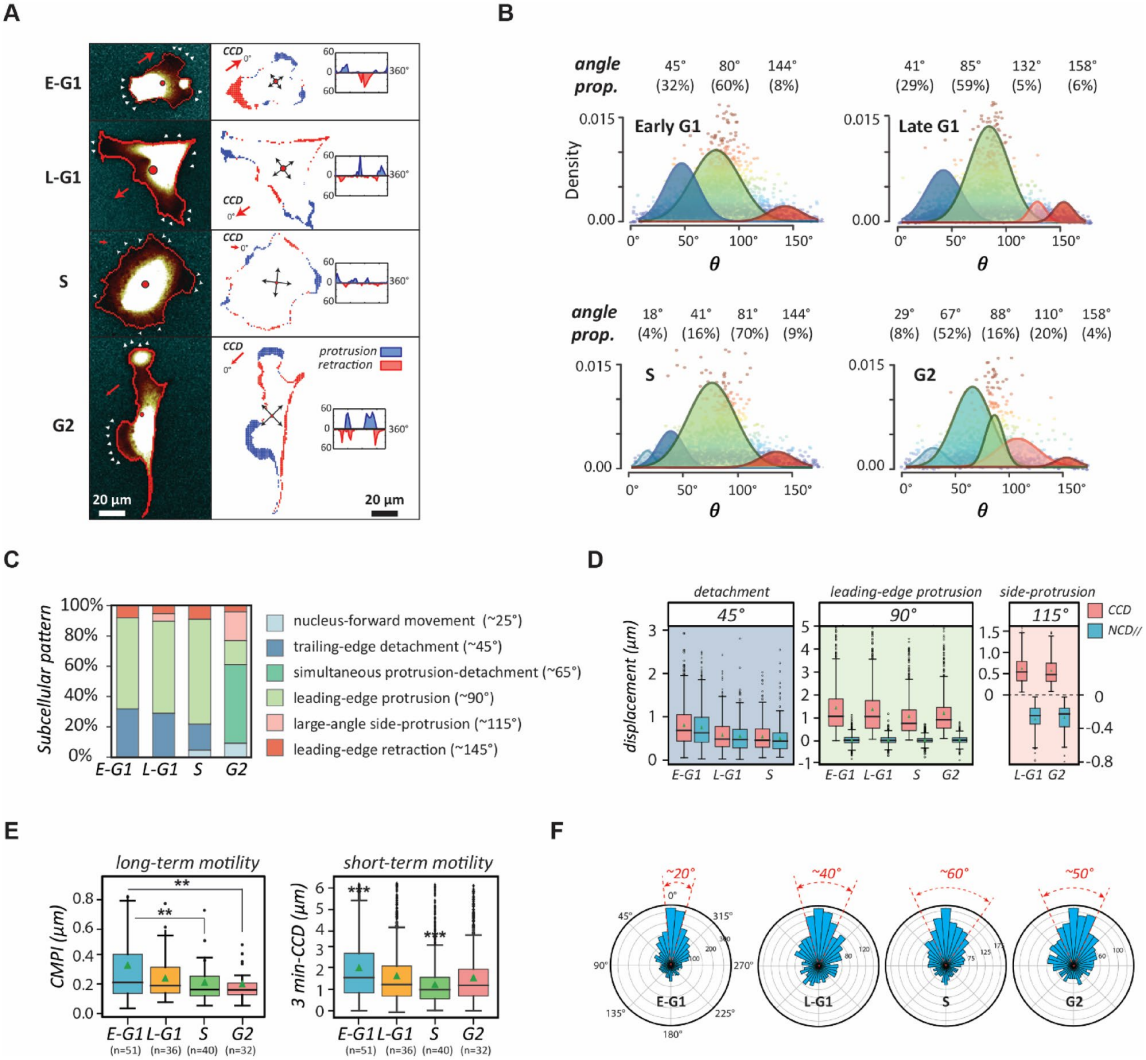
The transition of natural migration patterns of NIH 3T3 fibroblasts over the cell cycle. (**A**) Graphic of typical spreading patterns of NIH 3T3 fibroblasts in each stage of the cell cycle phases. Cell migratory dynamics can be assessed by peripheral variations in the polar coordinate in which cell centroid is set as the origin—left column: fluorescent cell images in different stages of the cell cycle phases; right: the occurrences of protrusions (blue) and detachments (red) in the corresponding synchronized cell edges; insert: the distribution of protrusion and retraction. (**B**) The decomposed UNM signature plots of synchronized cells showing cell migration patterns in different stages of the cell cycle phases, where the peak polar angle of each normal distribution represents a distinct subcellular activity with a specific weight. (**C**) Cell migration patterns of NIH 3T3 fibroblasts decomposed into different subcellular events. (**D**) Box plots of CCD and NCD_//_ in each signature polar angle zones, representing the magnitude of distinct subcellular events. (**E**) Cell motilities in different stages of the cell cycle phases, estimated using the Cell Migration Potential Index (CMPI) for the long-term and three-minute CCD speed for the short-term. (**F**) Cell migration polarity in different stages of the cell cycle phases, evaluated by the one-minute CCD direction histogram. Data information: *** indicates that the group of data is statistically significant compared to other groups. The related statistical results of this figure are listed in Supplementary Table 2.

To clarify the role of the cell cycle progression in driving cell phenotype transformation, we first assessed the cell dynamics that results in appearing cell morphology in every interphase stage of the cell cycle phases. We acquired cell images of the same cell under 3-min intervals and compared them through every 10° of cell boundaries to characterize the changes in cell morphology. The results reveal the dynamic status of the cells in each stage of the cell cycle phases, as shown in the representing cells (Fig. 3A). Cells in the early G1 phase are highly motile and move along a consistent direction; however, this consistency gradually diminishes during the late G1 phase. In the S phase, cells are relatively still with the peripheral ruffling in random directions. Interestingly, the cells in the G2 phase cell motion resurges by notable side-protrusions (*i.e.*, these motions do not align along a fixed axis).

Consequently, we asked how these short-term cell dynamics transform into long-term cell motility. The *CN correlation* analysis^26,27^ was applied to the movies of single NIH 3T3 fibroblasts, synchronized within each stage of the cell cycle phases, to systematically characterize their migration patterns (Fig. 3B & Table 1). This analysis not only specifies unique motion patterns by the cell cycle stages but also illustrates the evolution of phenotypic transition of cells throughout the cell cycle (Fig. 3C).

**Table 1:** The proportions of subcellular activities contributed to the cell migration patterns of cells in different cell cycle phases.

The cell cycle phase	Early G1	Late G1	S	G2
~ 25°: nucleus forward movement	–	–	18° (4%)	29° (8%)
~ 45°: trailing-edge DE	45° (32%)	41° (29%)	41° (16%)	–
~ 65°: simultaneous DE-PRO	–	–	–	67° (52%)
~ 90°: leading-edge PRO	80° (60%)	85° (59%)	81° (70%)	88° (16%)
~ 115°: side PRO	–	132° (5%)	–	110° (20%)
~ 145°: leading-edge retraction	144° (8%)	158° (6%)	144° (9%)	158° (4%)

The table listing the subcellular activities with signature polar angles and their percentiles in each stage of the cell cycle phases, determined by the *CN correlation* analysis. DE and PRO denote detachment and protrusion, respectively.

In the early G1 phase, cells exhibit a standard, highly polarized fibroblast behaviour, where detachment events take ~ 30% of the time and protrusion events take ~ 60% (Fig. 3D). In the late G1 phase, the cells deviate from the initial directional migratory mode and their polarity fades, judging by the increase of side protrusion events (from none to 5%) and the reduction of *CCD* and *NCD*_//_. In the S phase, the detachment events drop significantly to 16%, while the protrusion events are dominant at 70%. Meanwhile, the sizes of *CCD* and *NCD*_*//*_ are both at a minimum, indicating the cells have lost their polarities and intend to remain stationary. Notably, upon reaching the G2 phase, the cells only perform pure protrusion 16% of the time. In contrast, 52% of the time the cells are predominantly occupied by simultaneous detachments and protrusions (also see Table 1). In addition, the cells also exhibit large-angle side protrusion 20% of the time. This irregular dynamic pattern is illustrated by the G2-cells shown in Fig. 3A, where the cell displays neither a polarized nor isotropic, but rather, an irregular shape.

We then estimated the cell motility in each stage of the cell cycle phases using *CMPI* values. The results suggest that the motility of the cells in the G2 phase is low, comparable to in the S phase (Fig. 3E). However, the instantaneous speeds (3-min *CCD*) of the cells in the G2 phase are relatively high and similar to those in the late G1 phase. Hence, the high instantaneous speeds are not translated to the long-term motility for the G2-phase cells, a typical phenomenon for poorly polarized phenotypes. This conclusion is further supported by the persistency evaluation (Fig. 3F), which shows that the *CCD* directions alter more frequently for the cells in the G2 phase than in the G1 phase. It is noteworthy that the *CN correlation* analysis is highly consistent^27^. The convergence analysis here verified the homogeneity of cell migration patterns within each stage of the cell cycle phases (Fig. 3D & Supplementary Fig. 2). Since the cell morphology and dynamics are both governed by the stages of the cell cycle, these results strongly suggest that the heterogeneity of cell phenotype in the same cell type originates from the distribution of the cells in different stages of the cycle phases. In addition, the tight connection between the cell dynamics and the appealing morphology supports that the outcomes of cell dynamic measurements can present the phenotype heterogeneity.

### The actin-cytoskeletal remodeling leads to the cell-cycle-dependent migration pattern

**Figure 4:**
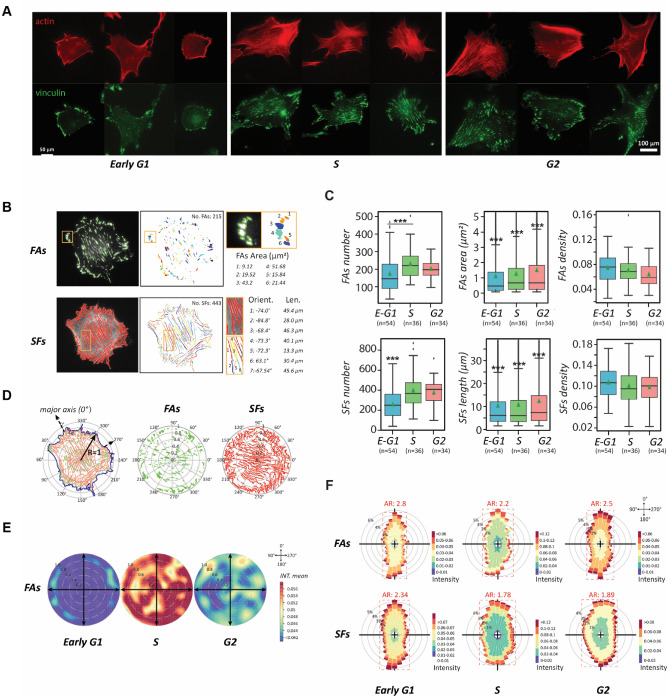
The transition of cell motility and polarity through the cytoskeletal remodeling over the cell cycle. (**A**) The cytoskeleton (actin; red) and adhesion plaques (vinculin; green) in NIH 3T3 fibroblasts over interphase of the cell cycle. Scale bar: 100 µm. (**B**) Segmented focal adhesions (FAs) and stress fibers (SFs) subjected to quantitative analysis using computer vision–left column: visualization of boundary segmentation of fluorescent cell images; middle: quantity of segmented FAs and SFs in single cells, shown in different colors; right: the quantification of FA and SF features, including size, length, and orientation. (**C**) Box plots presenting FAs and SFs dynamics during the cell cycle progression using the total number, single FAs area/SFs length, and density over the cell body. (**D**) The localization distribution of FAs in the cell normalized to a circle, where the distance to the centroid and the closest edge counts serve as the normalized position and the distribution is quantified by polar coordinates, in which cell centroid is set as the origin, and the 0° is aligned to the major axis. (**E**) Rose plots showing the distribution of FAs in each cell cycle stage at the cell population level. The degree of mean intensity is represented by warm (active)-cool (non-active) colormap. (**F**) Polar histogram plots showing the frequency localization of FAs (up) and SFs (down) of each stage of the cell cycle phases, where AR denotes the aspect ratio of the frequency distribution shape. The greater value of AR represents a more polarized FAs/SFs distribution. Data information: *** indicated that the group of data is statistically significant compared to other groups. The related statistical results of this figure are listed in Supplementary Table 3.

After every phase transition, the cells transformed into another morphology with new dynamics. As such, we continued evaluating how these dynamic processes occur. Cell dynamics are controlled by cytoskeletal remodeling. Migration-related subcellular activities, including protrusion, detachment, contraction, adhesion, etc., all stem from spatiotemporally assembling and disassembling of actin filaments^30,31^. Hence, we examined the actin-cytoskeleton and cell adhesions of the canonical NIH 3T3 fibroblasts in different stages of the cell cycle phases through fluorescence microscopy (Fig. 4A). The representing micrographs show that the early G1-phase cells have an amorphous actin-network and sparsely distributed adhesion plaques, which hallmarks the motile pattern^32,33^. In contrast, in the S and the G2 phase, cells form a defined cytoskeleton with highly organized stress fibers and dense-patched cell adhesions, signifying a stable, immotile pattern^34^.

To study the cell-cycle-phase-dependent migration patterns further, we scrutinized the stress fibers (SFs) and focal adhesions (FAs) in detail. The number, size (area), and density of the SFs and FAs were measured from the corresponding cell images (Fig. 4B), providing insightful cytoskeletal evolution throughout the cell cycle (Fig. 4C). In the early stage of the G1 phase, small adhesion plaques are scattered over the whole cell bodies. These adhesion plaques are not matured and could be disassembled easily; thus, the cells are highly motile^35^. In the S phase, the overall numbers and sizes of both FAs and SFs increase in a cell, but their densities drop over the enlarged cell body, indicating that the adhesion plaques are merged into matured patches and the extended SFs become stable, which lead to immotile cells. Even though the G2-phase cells continuously possess thick SFs and matured FAs from the S phase, the density of FAs is significantly reduced. This change inspires the speculation for the occurrence of focal adhesion turnover.

To clarify whether focal adhesion turnover had occurred, we analyzed individual cells to normalize their cell shape and size dependence and statistically characterized the FAs within the cells (Fig. 4D). We utilized the polar coordinate system, set the origin at each cell’s centroid, the major axis of the cell in the direction of the leading edge as polar angle 0˚ for each cell, and the radii as the normalized distances from the cell centroid to the radiated cell edges. Following this format, FA distributions in different cells were overlaid on a rose plot with a colormap representing the density (Fig. 4E). The rose plots demonstrate that cells in the S phase possess the highest density of adhesions. Therefore, the comparison of local adhesion densities between cells in the S and the G2 phase supports the idea that the FAs diminish in certain regions of the G2 cells and focal adhesion turnover occurs.

The occurrence of focal adhesion turnover provides a rational justification for the 52% of prevalent “unconventional” cell motion and 20% of side protrusion, deciphered by the *CN correlation* analysis. As a result, parts of the cell periphery are freed up so that pseudopods can form and give rise to an irregular morphology and limited local migration. This type of migration shifts the cells locally in a random direction and is highly dynamic with the cell instantaneously speed comparable to the cells in the G1 phase.

To study how the cytoskeletal features impact cell polarity over the cell cycle, we also analyzed the distributions of FAs and SFs through their polar histograms in different stages of the cell cycle phases (Fig. 4F). After the polar histograms were determined through the rose plots with 10˚ resolution, the data showed that both distributions are anisotropic in all cell cycle phases. The degree of isotropy can be determined by the aspect ratio (AR) of the histograms, where a greater value indicates that the probed cytoskeletal features are distributed with more bias, leading to a strong polarity. The results reveal the G1-phase cells have the highest AR value, followed by the G2-phase cells, then the S-phase cells with the lowest AR value. Thus, the polar histogram analysis restates that cells lose polarity when approaching the S phase, and regain some polarity in the G2 phase through focal adhesion turnover. Since cytoskeleton remodeling is the driving machinery of cell morphological dynamics, our results reiterate the postulation that a good portion of cell heterogeneity originates from the subpopulations of cells distribute in different stages of the cell cycle phases.

### Two CDKIs—p27^Kip1^ and p21^Cip1^ guide the RhoA and Rac1 signaling in migration evolutions

The alteration of cell phenotypes across cell cycle stages should be inexorable from a molecular-level perspective. Under such a condition, there must be certain molecules that connect the cell cycle to cell dynamics beyond Rho GTPases, the primary molecular switches for cytoskeletal remodeling^10^. These molecules must be associated with regulators of the cell cycle, the cyclin-dependent kinases (CDKs)^6,7^. To our best knowledge, the only known molecules directly interact with both Rho GTPases and the CDKs are two CDKIs: p27^Kip1^ and p21^Cip1^. After cells entering the S phase, p27^Kip1^ is exported from the nucleus to the cytoplasmic region to bind and inhibit RhoA, while p21^Cip1^ binds and downregulates the ROCK^13,14^.

**Figure 5:**
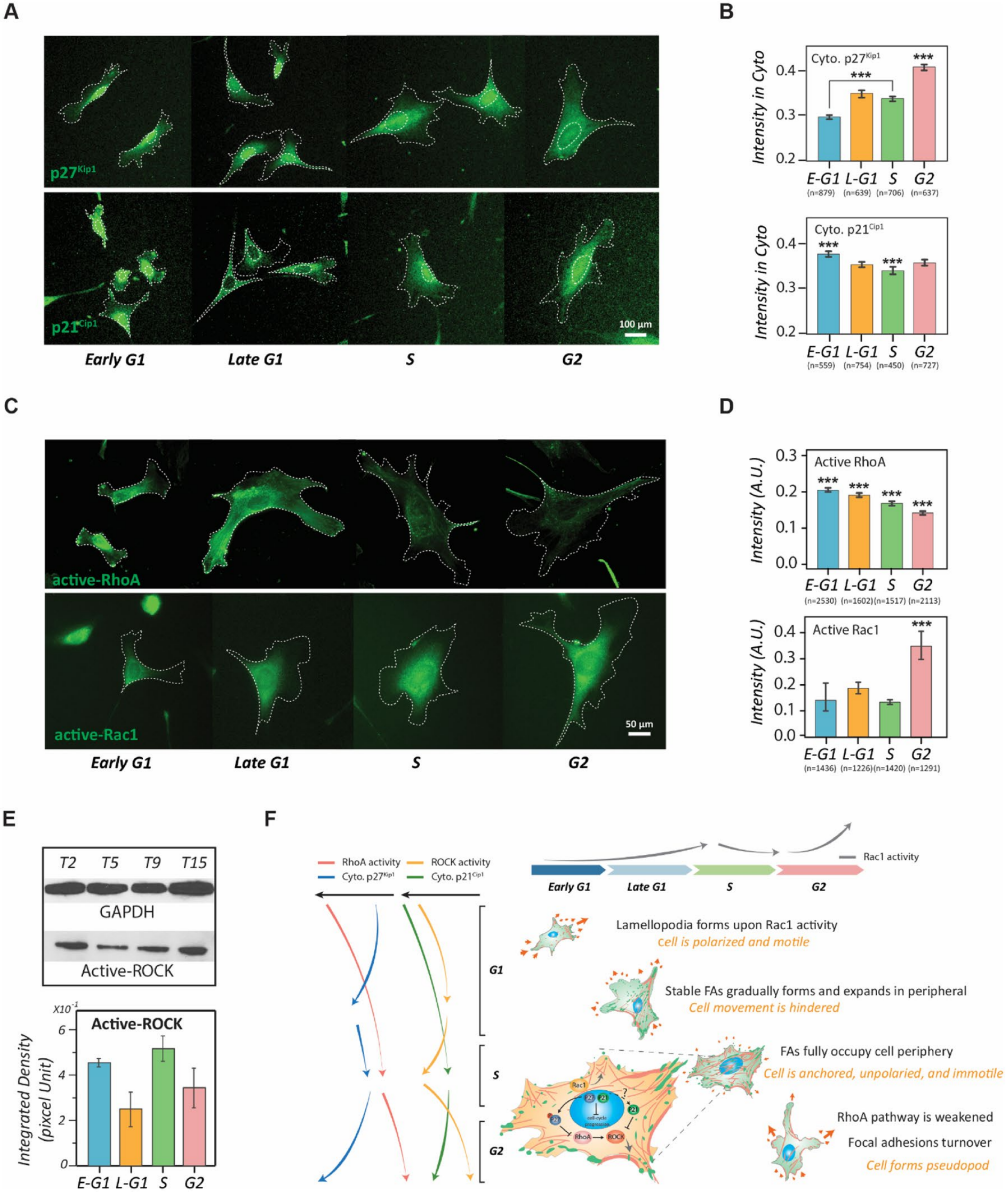
The involvement of cytoplasmic p27^Kip1^ and p21^Cip1^ in regulating the cell-cycle-dependent cell phenotype transitions. (**A**) Fluorescent images of p27^Kip1^ and p21^Cip1^ in each stage of the cell cycle phases. Dotted lines depict the boundaries of the cells and nuclei. Scale bar: 50 µm. (**B**) Bar plots showing the concentration profiles of the cytoplasmic p27^Kip1^ (up) and p21^Cip1^ (down) in different stages of the cell cycle phases. Error bars represent the SEM. (**C**) Immunostaining of active-RhoA and active-Rac1 in NIH 3T3 fibroblasts of each stage of the cell cycle phases. Dotted lines depict the boundaries of the cells. Scale bar: 50 µm. (**D**) Bar plots showing the activity profiles of active RhoA (up) and active Rac1 (down) in single cells of different cell cycle stages. (**E**) Western blot results of active ROCK in different cell cycle phases. Control: GAPDH. Error bars represent standard errors (n = 2). (**F**) Metaphorical landscape illustrating the migration phenotype transitions caused by Rho-GTPase and CDKIs pathways along with cell cycle progression. The magnified view of the S-phase cells shows the molecular interactions between CDKIs and RhoA pathways in the S phase. Data information: *** indicates that the group of data is statistically significant compared to other groups. The related statistical results of this figure are listed in Supplementary Tables 4 and 5.

To observe whether their spatiotemporal appearances agree with the activity profiles of RhoA and Rho kinase (ROCK) throughout the interphase of the cell cycle, we monitored the distributions of p27^Kip1^ and p21^Cip1^ in cells of different cell cycle phases. p27^Kip1^ and p21^Cip1^ were immuno-stained in the synchronized NIH 3T3 fibroblasts of different cell cycle stages to quantitatively determine their cytoplasmic concentration profiles (Fig. 5A). The results shows that the cytoplasmic concentration of p27^Kip1^ elevates along the cell cycle progression, while cytoplasmic p21^Cip1^ exhibits a mild concave evolution with the lowest concentration appearing in the S phase (Fig. 5B). Consequently, we conducted quantitative fluorescence microscopy to determine the activity profiles of RhoA and Rac1, the regulators of actomyosin contraction and membrane protrusion, respectively^36^ (Fig. 5C). The results show that the RhoA activity monotonically drops throughout the interphase of the cell cycle; whereas the Rac1 activity slightly varies from the early G1 phase to the S phase, but elevates significantly to a maximum in the G2 phase (Fig. 5D). The trends of these profiles are supported through the RhoA and Rac1 pull-down/Western blot assessments (Supplementary Fig. 4). We also conducted Western blotting against phosphorylated myosin phosphatase target subunit 1 (MYPT1, Thr696), which indicates the ROCK activity, to determine the activity profile of ROCK in the interphase of the cell cycle (Fig. 5E). The results show that the ROCK activity significantly reduces after entering the G2 phase, echoing the p21^Cip1^ increasing in the cytoplasmic region and the diminishing of RhoA activity.

The comparison of these profiles shows that a spatiotemporal agreement for the intermediate roles of p27^Kip1^ and p21^Cip1^ in the crossroad between the cell cycle and the cell movement. The increase of cytoplasmic p27^Kip1^ and p21^Cip1^ in cells from the S phase to the G2 phase is correlated with the decrease of RhoA and ROCK activities, respectively. Since the inhibition of the RhoA pathway leads to the disassembly of focal adhesion^37^, the weakening of RhoA pathway activity in the G2-phase cells explains the concurrence of focal adhesion turnover as we observed. In addition, the depleting of RhoA pathway activity only occurs after the cells entering the S phase where the p27^Kip1^ and p21^Cip1^ are released from the CDK-cyclin complex and become cytoplasmic^13–16,38^.

### The brief cell-cycle-phase-dependent cell morphology model

Immediately after cytokinesis, a newly divided cell enters the early G1 phase and spreads on the extracellular matrix (ECM), the cell explores its microenvironment via lamellipodia, which are formed upon the Rac1 activity. In this period, the cell is also polarized along with the ECM that offers dense anchorage sites. Meanwhile, in the presence of mitogen, RhoA is activated, further promoting focal adhesions maturation in the polarized sites^36,39,40^. Eventually, the formation of dense, stable focal adhesions causes the hindrance of cell movement and the cell reaches the late G1 phase. During this time, the migration retention leads to prolonged substrate contacts around the cell periphery, inducing lateral protrusions through the activation of proximal Rac1 activity. When the cell periphery is fully occupied by stable adhesions, the cell becomes stationary and enters the S phase. Starting around the same time, the presence of cytoplasmic CDKIs gradually diminishes the RhoA pathway activity, eventually causing focal adhesion turnover in the G2 phase. Hence, some parts of the cell periphery are freed up again and available for new cell-substrate contact developments, allowing the cell to form pseudopod-like features through re-surged Rac1 activity. This cytoskeletal remodelling alters cell morphology to be a form suitable for mitosis. Together, the evolution of cell phenotype is highly connected to the cell cycle progression that renders the fact that cell cycle stages can be considered as subpopulations of cell heterogeneity with distinct functional significance (Fig. 5F).

## Discussion

This study reveals that both the morphology and dynamics of NIH 3T3 fibroblasts evolve along the interphase of the cell cycle with distinct molecular mechanisms. In the G1 phase, the most motile stage, the RhoA pathway plays an active role in promoting cell migration, while in the G2 phase, another dynamic interphase, the RhoA pathway becomes passive and CDKIs play a pivotal role in inhibiting the RhoA pathway and contributing to focal adhesion turnover to guide cytoskeletal remodeling. Furthermore, these results also allow us to hypothesize that, in addition to the existence of intratumor heterogeneity, cell-cycle-mediated drug resistance also allows some cancer cells to survive from the otherwise effective drugs and complicates prognostics^41–43^. Hence, this study aims to avoid the confounding factors in heterogeneity caused by the cell cycle through suggesting a valuable strategy to evaluate the specific variables underlying drug resistance issues when applying target drugs in fighting cancers, making it necessary to take the molecular mechanisms and causes of heterogeneity into account.

We made these conclusions based on three criteria: First, statistical evaluations reveal that all cell phenotypes, including motility, polarity, and morphology, maintain a high level of homogeneity in each stage of the cell cycle. The uniformity of these cellular behaviours in individual cell cycle phases is critical for the construction of the cellular/molecular framework. Second, the *CN correlation* approach allows us to decipher the cell dynamic patterns (*i.e.*, the quantitative contributions of different subcellular activities in the whole cell motion process) of single cells in different stages of the cell cycle. Finally, since the molecular mechanisms of individual subcellular activities in cell dynamics have been clearly determined^31,36,44^, the mechanisms of these subcellular activities have laid the foundations to thoroughly connect the activity profiles of the controlling molecules to the cell phenotype transitions over the cell cycle phases.

Despite the activity profiles of key proteins tightly correspond to a specific cell behaviour, the whole cell physiology cannot be solely determined by discrete genomic and proteomic data due to the complex signalling crosstalk. However, the abundance of molecular information can be tightly integrated by single-cell behaviour analysis platform, such as through the *CN correlation* analysis^26,27^, which collectively describes live-cell behaviours and their subcellular dynamic information in depth. A single-cell behaviour analysis platform should be able to generate activity profiles from selected cell behaviour with high sample sizes for statistical evaluations, therefore providing high-resolution information regarding the weighing contributions of composed subcellular activities. This capacity would allow the extension of applying machine deep learning into cell physiology.

## Materials and methods

### Cell preparation

NIH 3T3 fibroblasts (American Type Culture Collection, Manassas, VA) were cultured in DMEM (Mediatech, Manassas, VA), supplemented with 10% FBS (Hyclone Laboratories, Logan, UT), 1% L-glutamine (Mediatech), and 1% penicillin–streptomycin (Mediatech). Cells were maintained in an incubator with 10% CO_2_ and at 37 °C. The cell cultures were also kept below 70% of confluency. For image acquisition, cell samples were prepared on the glass bottom dishes, which were pre-treated with 0.01% poly-L-lysine (Sigma-Aldrich, St Louis, MO), following by 20-µl/ml fibronectin (BD Biosciences, San Jose, CA) coating.

### Cell synchronization

Cells were synchronized in the cell cycle using a double thymidine approach^45^. Two-mM thymidine (Sigma-Aldrich) was applied to the culture medium for 12 h to arrest the cells at the stage of the G1/S phase transition. Then, the cell culture was gently washed sequentially by HBSS (Mediatech), DMEM, and fresh culture medium, each for 15 min. Afterwards, cells were kept in regular culture medium for 9 h before another 12-h thymidine arrest. Finally, the cells were subjected to the same washing procedure described above to release the cells from cell cycle arrest. After the cells were released, the cell cycle profiles were determined hourly through individual cultured batches (see next section).

### Cell cycle profile determination using fluorescence microscopy

With the sample size of ~ 5000 cells, the nuclear intensity histogram was constructed using 50 bins and subjected to the Dean-Jett-Fox model^46,47^ to determine the profile of the cell cycle phases. Briefly, normal curves with the same coefficient of variations were individually fitted to the G1 and the G2/M peaks of the histogram using least-squares approximation. Then, the superposition of a normal curve and a broadened second-order polynomial was fitted to the histogram associated with the S phase. The areas covered by individual curves were used to determine the percentages of cells associated with the corresponding phases of the cell cycle.

### Cell cycle profile determination using flow cytometry

Individual batches of cell samples were detached from the culture dishes by trypsin and subjected to centrifugation. Then, the pellet was gently rinsed 3 times using ice-cold PBS before being fixed by 70% ethanol overnight at 4 °C. The next day, the pellet was re-suspended in PBS that contains 100-μg/ml ribonuclease, incubated for 30 min at room temperature, and subjected to 50-μg/ml propidium iodide (Abcam, Cambridge, MA) to label the DNA for 30 min at room temperature. Afterwards, LSR-II flow cytometer (BD Biosciences) and FACSDiva (BD Biosciences) were used to determine the percentage of cells in each phase of the cell cycle.

### Live-cell labelling

pEGFP plasmid (BD Biosciences) was transfected into NIH 3T3 fibroblasts using lipofectamine reagent (Invitrogen, Carlsbad, CA) based on a standard protocol. After transfection, the cells were cultivated onto the glass bottom dishes (World Precision Instrument, Sarasota, FL) by the single-cell density. After 24 h, the cells were fluorescently labeled with GFP. Ten minutes before image acquisition, 20-µg/ml Hoechst 33,342 was applied to the cell culture to label the nuclei^48^.

### Fixed cell labelling

Fibroblasts were fixed by 4% paraformaldehyde (Sigma-Aldrich) in PBS for 15 min, permeabilized by 0.25% Triton X-100 (Sigma-Aldrich) in PBS for 10 min, and blocked by 5% BSA (Sigma-Aldrich) in PBS for 1 h. For immunofluorescence staining, cells were incubated with primary antibody (listed in Supplementary Table 6) overnight at 4 °C and washed 3 times by PBS. Then the corresponding fluorescently labeled secondary antibody was applied for 1 h at room temperature before PBS washes. For F-actin and DNA staining, cells were incubated with 1:40 dilution of Alexa Fluor 568-phalloidin (Invitrogen) and 0.1-μg/ml Hoechst 33,342. These stains were loaded with secondary antibodies at the same time.

### Microscopy and image acquisition

Fluorescent images were acquired using a TE-2000E imaging acquisition system (Nikon, Melville, NY), equipped with a 20 × objective lens, a 60 × oil-immersion objective lens, a digital eclipse C1 laser scanning confocal module, an X-Cite 120 PC fluorescent light source (EXFO, Ontario, Canada), and a Cascade: 1 K CCD camera (Roper Scientific, Tucson, AZ). To avoid photobleaching, acquisition parameters were set at 300-ms exposure time, 3 × 3 binning, and 25% maximal light intensity. All acquired signals were not saturated under such a setting. During live-cell image acquisition, samples were placed in a CO_2_ supplementary system (In Vitro Scientific, St. Louis, MO), maintained at 10% CO_2_ and 37 °C. Live-cell movies were recorded for 1 h through two-channel fluorescence microscopy (the green channel for cells and the blue channel for nuclei) at 1-min time intervals. Fixed fluorescent beads were bombarded on the glass-bottom slide during sample preparation to serve as the immobile references for image analysis to minimize positioning errors^49,50^.

### Rho GTPases pull-down assays

pGST-Rhotekin-RBD and pGST-PAK1-PBD plasmids (Addgene, Watertown, MA) were separately transformed into bacteria to produce recombinant fusion proteins. Afterwards, glutathione Sepharose 4B resin (GE Healthcare Life Sciences, Pittsburg, PA) was added to the bacterial lysate for centrifugation. Then, the pellet was re-suspended in PBS with 10% glycerol, stored at − 80 °C in 20-μl aliquot, and used as baits later for active RhoA and active Rac1 pull-down assay, respectively^51^. Before pull-down, the aliquot was quantified using BSA as standard for consistency. Cell lysates were prepared in RIPA lysis buffer (10-mM Tris HCl, pH 7.4, 150-mM NaCl, 1% (v/v) Triton X-100, 0.1% (v/v) SDS, 0.5% (v/v) DOC and 1-mM EDTA), containing protease inhibitor cocktail (Cytoskeleton, Inc., Denver, CO), and cleared by centrifugation (20,000 × g). Then, each lysate sample was mixed with an equal amount of pull-down resin and incubated at 4 °C overnight. The next day, the sample was pulled down by centrifugation, washed 3 times by lysis buffer before the resin-bonded protein was dissociated by 2 × SDS sample buffer for 2 min at room temperature. The sample was then boiled for 5 min, and subjected to 12% SDS PAGE, following by Western blotting.

### Western blotting

After SDS-PAGE, the samples were transferred to the nitrocellulose membrane. Then, the membrane was blocked by 1X TBST with 5% w/v nonfat dry milk for 1 h at room temperature, and incubated with the primary antibody against the target protein overnight at 4 °C. The next day, the membrane was washed with TBST, and incubated with horseradish-peroxidase-conjugated secondary antibody for 1 h at room temperature. Detection was made by a standard protocol. The blotting results were quantified by ImageJ (NIH, Bethesda, MD). Antibodies are listed in Supplementary Table 6.

### Quantitative PCR to measure mRNA level

The total RNA in the cell sample was isolated by the RNeasy Mini Kit (Qiagen, Valencia, CA) and reverse-transcribed to cDNA by the iScript™ Advanced cDNA Synthesis Kit (Bio-Rad, Hercules, CA). Then, the quantities of cDNA were determined through quantitative PCR (qPCR) using the CFX connect system (Bio-Rad). qPCR reactions were conducted using the SYBR supermix (Bio-Rad). The GAPDH quantities in cell samples were treated as the standard for cross-comparison. Intron-spanning primers for RhoA (JN971019.1), Rac1 (NM_009007.2), cyclin A1 (BC120518.1), cyclin E1 (BC138662.1), and GAPDH (GU214026.1) were designed using Primer3 and synthesized (Integrated DNA Technologies, Skokie, IL) as follows:

**Table:** 

mRNA	sense	antisense
RhoA	AAACAGGATTGGCGCTTTTG	CACAAGATGAGGCACCCAGA
Rac1	AAGTGTGTGGTGGTGGGAGA	GTCAAAGACGGTGGGGATGT
Cyclin A1	AGTACCTGCCTTCACTCATTGCTG	TCTGGTGAAGGTCCACAAGACAAG
Cyclin E1	GCCCTCTGACCATTGTGTCC	GCACCACTGATAACCTGAGACCT
GAPDH	TCTCTGCTCCTCCCTGTTCC	GTTCACACCGACCTTCACCA

Measurements of each protein sample were conducted through 2 experimental duplicates and 2 biological duplicates. Relative cDNA levels were determined using the comparative Ct method^52^.

### Statistical analysis

For live single-cell phenotype analysis, the sample sizes were greater than 20 cells. For fixed single-cell analysis, the sample sizes ranged from 200 to 2000. Data were displayed as mean ± SEM in either bar plot or box plot. The analysis of variance (ANOVA) was applied to compare the means of a condition among groups (more than 2 groups). When multiple group comparison by ANOVA showed significant differences exist among the compared group, the Tukey's multi-comparison method would be applied to identify which groups are significantly different (*P* < *0.05*). The significant differences are labeled as *, **, and ***, to correspond to the *P* < 0.05, 0.01, and 0.001 criteria, respectively.

### The cell phenotype descriptors

The profiles of phenotype parameters were analyzed at 3-min intervals (non-overlaid) over the cell cycle. Three significant cell shape phenotype parameters^53^ were set as cell area, aspect ratio, and circularity. The area was identified by the actual number of pixels in the region times the scale unit per pixel^54^; the aspect ratio defined as the length of the major axis divides by the length of the minor axis; where the cell has been normalized as the ellipse; the circularity value is computed as ${\text{Circularity}} = 4 \times {\text{Area}} \times \pi /{\text{perimeter}}^{2}$.

### The partition of distinct data groups over the cell cycle progression

We applied the intrinsic function *lm* in R (RStudio, PBC, Boston, MA) to decide the partitions of the data group by ordinary least squares regression. The initial partitions were randomly picked for the given data set. The decision rule is

$$P = \left( {\left\langle {{\text{Group}}2} \right\rangle - \left\langle {{\text{Group}}1} \right\rangle } \right)^{2} \cdot \left( {\left\langle {{\text{Group}}3} \right\rangle - \left\langle {{\text{Group}}2} \right\rangle } \right)^{2}$$

where each <  > represents a mean of the divided subsection. At any time, the partitions of instantaneous speeds and the three shape parameters are the same during the iteration of the least-squares criterion.

### Homogeneity of cell long-term motility

To address the homogeneity of cell migration patterns within each cell cycle phase, we detected the convergence of *CMPI* value by continuously accumulating data by adding sample sizes until fulfilling the desired criterion (Supplementary Fig. 2). The analysis results reached a steady-state (the standard error of the mean is less than 10% of the mean value) once the sample size exceeded 10. The information illustrated that cell migration is highly homogeneous and predictable when cells were synchronized to a specific cell cycle phase, thus supporting the claim that cell migration underlies cell cycle progression.

### SFs and FAs localization analysis

To quantify the distribution of SFs and FAs, ~ 50 cells were collected at a certain condition for SF/FA localization analysis in the individual-cell level. The intensity distributions of SFs and FAs in different cells were normalized and overlaid to a polar coordinate. In this action, each cell centroid was set as origin, the orientation of the cell major axis was set as 0°, and a radius from each cell centroid to the radiated cell edges was set as 1 to describe the cell body (as seen in Fig. 4D). Then, *OpenAir* modules in the *R* platform were applied to visualize the localization profiles occurrence frequencies of the SFs/FAs. The *polarPlot* function displayed a bivariate polar plot of SF/FA densities in different locations. Intensity was calculated by the local mean values (Fig. 4E). The *polarFreq* function displayed the distributions and occurrence frequencies of SFs/FAs in all directions, where the scale shows the occurrence counts in each bin (Fig. 4F).

The computer vision approaches were performed by the MATLAB with Image Processing Toolbox (MathWorks, Natick, MA). The key approaches are described as following:

### Cell and nuclear boundary determinations

A robust Gaussian fitting algorithms^54^ was applied to determine the cell boundaries. The nuclear boundaries were determined by direct segmentation using a given intensity threshold.

### Relative protein amount quantification

Under appropriate fluorescence microscopy setting (*i.e.*, the intensity is not saturated), the amount of target protein was determined by the integrated greyscale value of the region of interest (*e.g.*, the cell, the cytoplasmic region, or the nucleus). The greyscale value was obtained from subtracting the background intensity by the mean intensity of the region of interest.

### UNM analysis

The occurrence diagram and *CCD* diagram of the *CN correlation* profile were de-convoluted into normal distribution curves through univariate normal mixtures (UNM) fitting in R (RStudio, PBC, Boston, MA). The best-fit model of UNM analysis was automatically achieved from the function *normalmixEM*, installed from package mixtools (R Foundation for Statistical Computing, Vienna, Austria. URL: https://www.R-project.org/).

### The analysis of SFs and FAs

Signals of SFs were subjected to a sequence of processes from raw images: (1) Get rid of irrelevant background noise using reduced Gaussian filter^54^. (2) Adjust the variance and magnitude of the signal using generic Gaussian and Laplacian filters. (3) Enhance crossing bright line using a linear Gaussian sensor^55^. (4) Conduct local binarization via a combination of Gaussian weighted adaptive means and global thresholding^55^. (5) Segment SFs mask to get a skeleton of binary SFs mask. (6) Identify branch points in a skeleton structure by MATLAB built-in function *bwmorph*. (7) Identify different branches of fibers as the residual images by subtracting branch points from the skeleton. (8) Trace and reconnect these segmented fiber-branches to enable reconstructed SFs (three basic criteria for connection procedure: the orientation of two compared fibers are less than 18°; the Euclidean maximum distance between two fiber branches endings are less than 8 pixels; and the mean intensity values and the width of fibers are to consistency, which is within 1.2 folds of difference) (Supplementary Fig. 3A).

Signals of FAs were subjected to a sequence of processes from raw images: (1) Get rid of irrelevant background noise using reduced Gaussian filter^54^. (2) Determine the local binarization by Otsu’s adjustable threshold method. (3) Apply morphological techniques called "opening-by-reconstruction" and "closing-by-reconstruction" to "clean" up the adjacent touching adhesion leagues. (4) Use a watershed segmentation approach to separate each FA (Supplementary Fig. 3B).

### Scientific diagrams

The data diagrams were originally generated by MATLAB (MathWorks, Natick, MA), Python (Python Software Foundation. Python Language Reference, version 3.6. Available at http://www.python.org), and R (R Foundation for Statistical Computing, Vienna, Austria. URL: https://www.R-project.org/). The pre-processing and quantification of Western Blots were achieved by ImageJ (Rasband, W.S., ImageJ, U. S. National Institutes of Health, Bethesda, Maryland, USA, https://imagej.nih.gov/ij/, 1997–2018). All schematic diagrams were hand-painted by Adobe Illustrator (Adobe Inc., 2019. Adobe Illustrator, Available at: https://adobe.com/products/illustrator).

## Supplementary Information

Supplementary Information.
