# Supplementary material for: Decomposition of cell activities revealing the role of the cell cycle in driving biofunctional heterogeneity
Source: Sci Rep. 2021 Dec 6;11:23431. doi: 10.1038/s41598-021-02926-4 (PMC8648726; doi:10.1038/s41598-021-02926-4)
Supplement: Supplementary file 1 — Supplementary Information. [file 41598_2021_2926_MOESM1_ESM.docx]

**Supplementary Information**


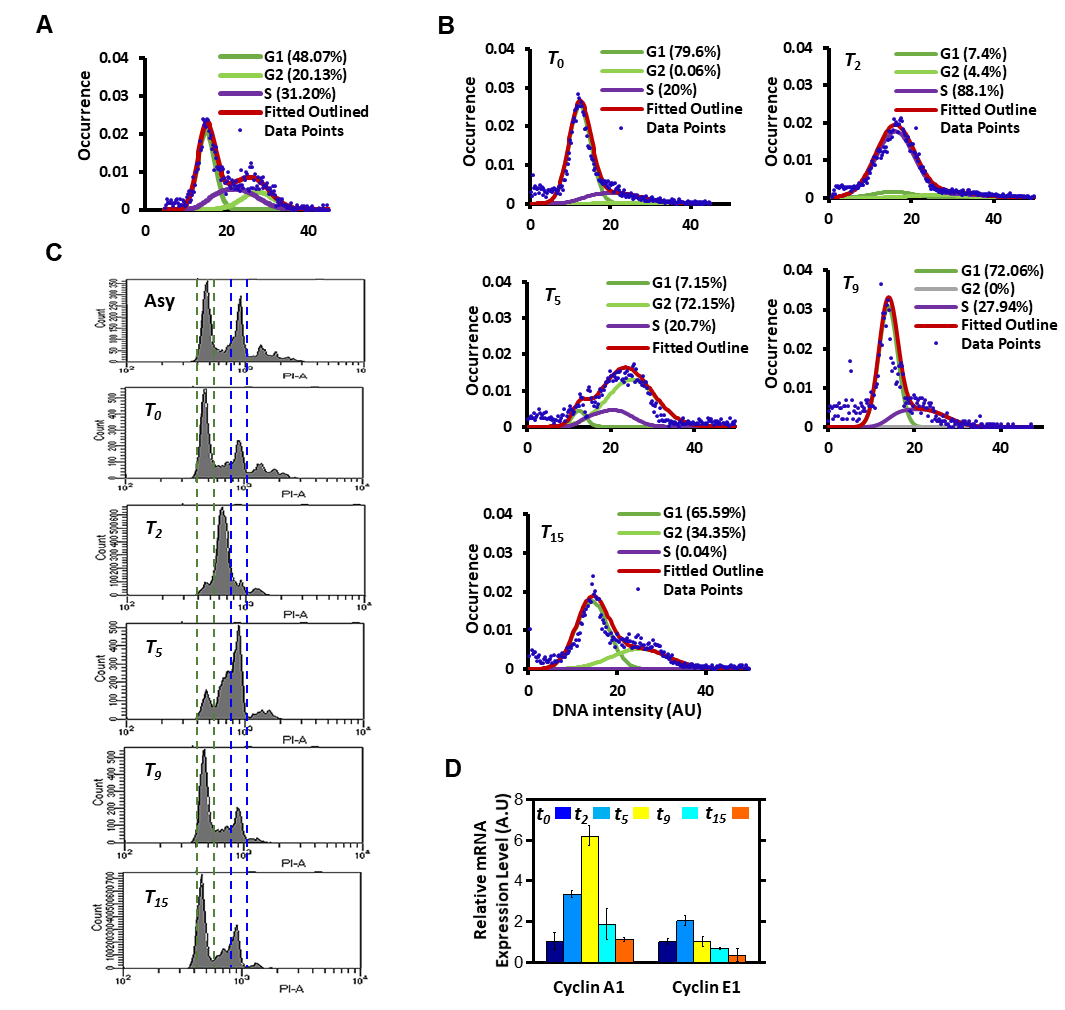
**Supplementary Figure 1. The cell cycle profile analyses of synchronized NIH 3T3 fibroblasts by time course, related to Fig. 1E.** (**A**) The nuclear intensity profile of normal, unsynchronized cells. The profile (red) was subjected to the modified Dean-Jett fitting model to determine the weight of the cell population in each cell cycle phase: G1 (orange), S (purple), and G2 (green) phase of the cell cycle. (**B**) The nuclear intensity profiles and Dean-Jett fitting model of synchronized cells after released from the cell cycle arrest for a certain time course. The time of the optimal profiles of synchronized cells propagate to the G1/S, the early S, the S, the G2, the early G1, and the late G1 phase were determined as 0, 2, 5, 9, and 15 hours, respectively (and denoted as T0, T2, T5, T9, and T15, respectively). (**C**) The profiles of the cell cycle phases at the same time courses of cell propagation, after the cells were released from the cell cycle arrest, were verified by flow cytometry. (**D**) The amounts of cyclin A and cyclin E cDNAs, quantified at T0, T2, T5, T9, and T15 (left to right) to verify the cell cycle synchronization. Error bars represent the standard deviation (n=2).

**
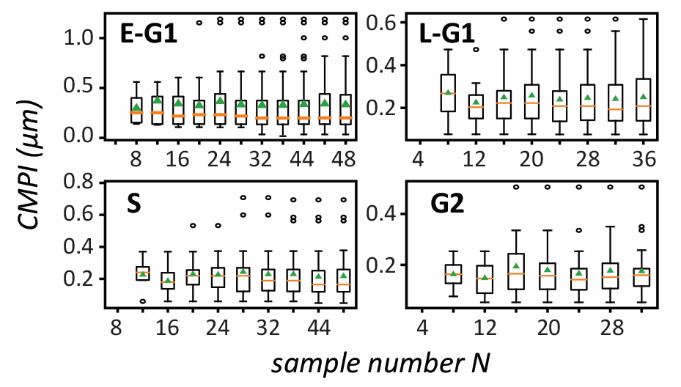
**

**Supplementary Figure 2. The convergence of long-term motility of cells in different cell cycle stages was estimated using *CMPI*.** Box plots showing the convergences of *CMPI* against sample size in different stages of the cell cycle phases. Error bars represent the standard error of the mean.


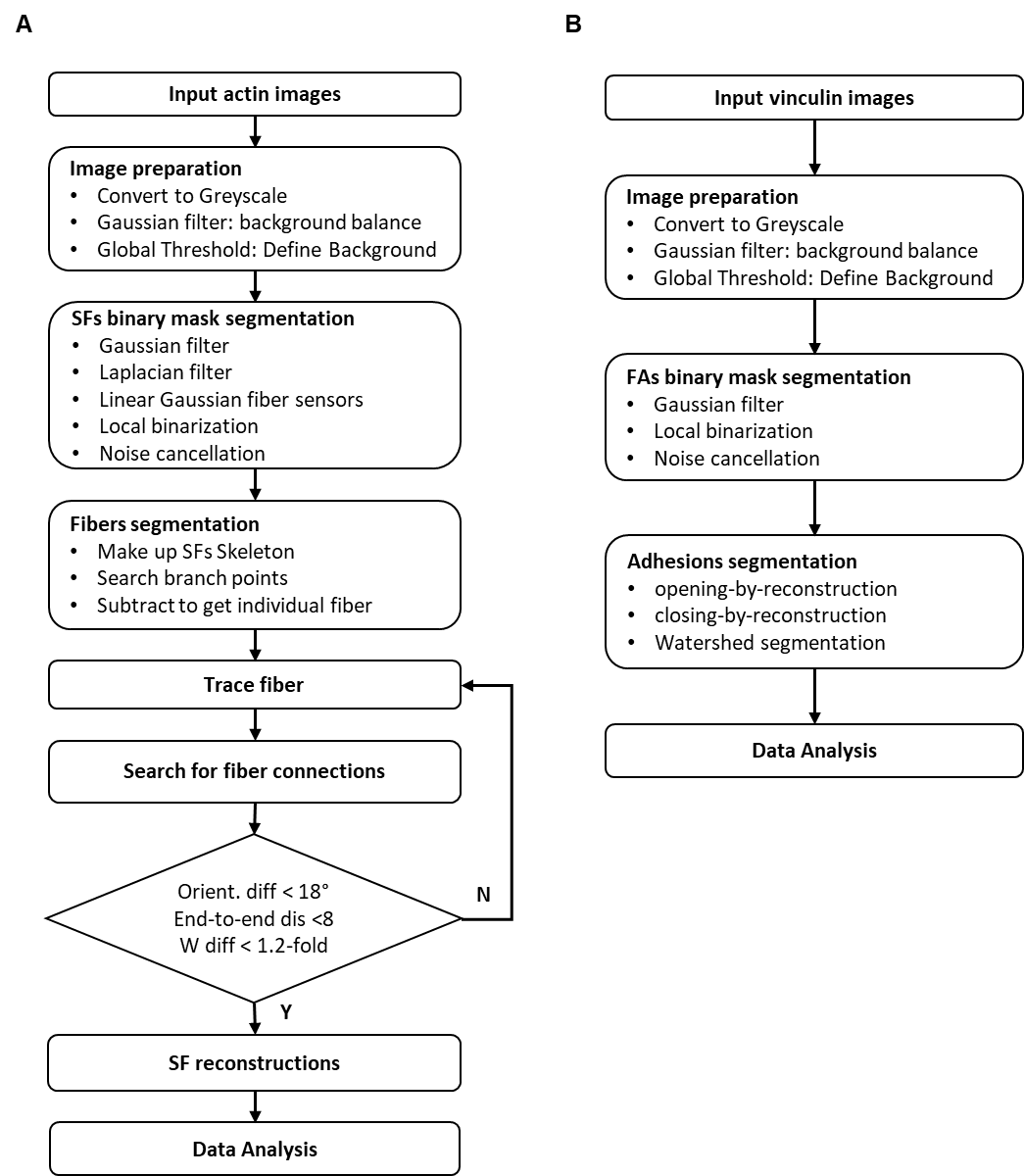


**Supplementary Figure 3. Flowcharts display the procedures of SF and FA analyses.** (**A**) The image processing procedure of SFs. (**B**) The imaging processing procedure of FAs.

**
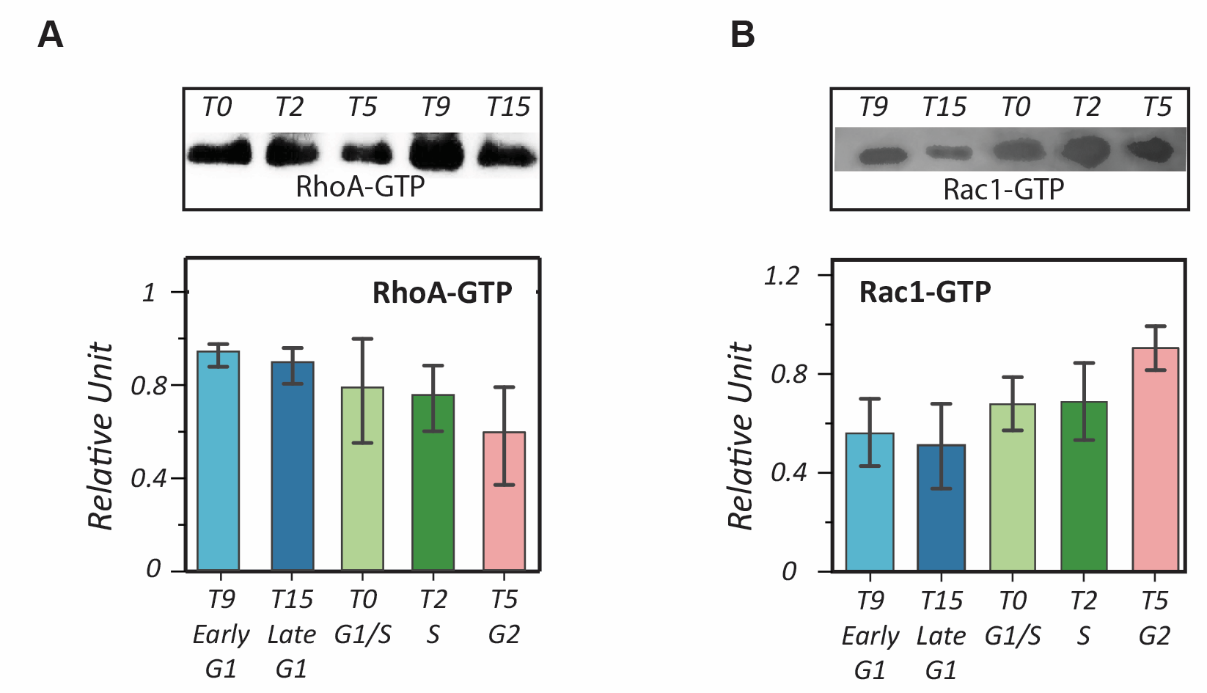
**

**Supplementary Figure 4. The activities and expression levels of RhoA and Rac1 in different cell cycle phases.** Activities of RhoA and Rac1, assessed by RhoA-GTP and Rac1-GTP pull-down assays, respectively, followed by Western blotting. (**A**) Western blotting results of the RhoA pull-down assay (upper panel; the full image is shown in Fig. S5B, First panel) in different stages of the cell cycle phases. (**B**) Western blotting results of the Rac1 pull-down assay (upper panel; the full image is shown in Fig. S5C, First panel) in different stages of the cell cycle phases.


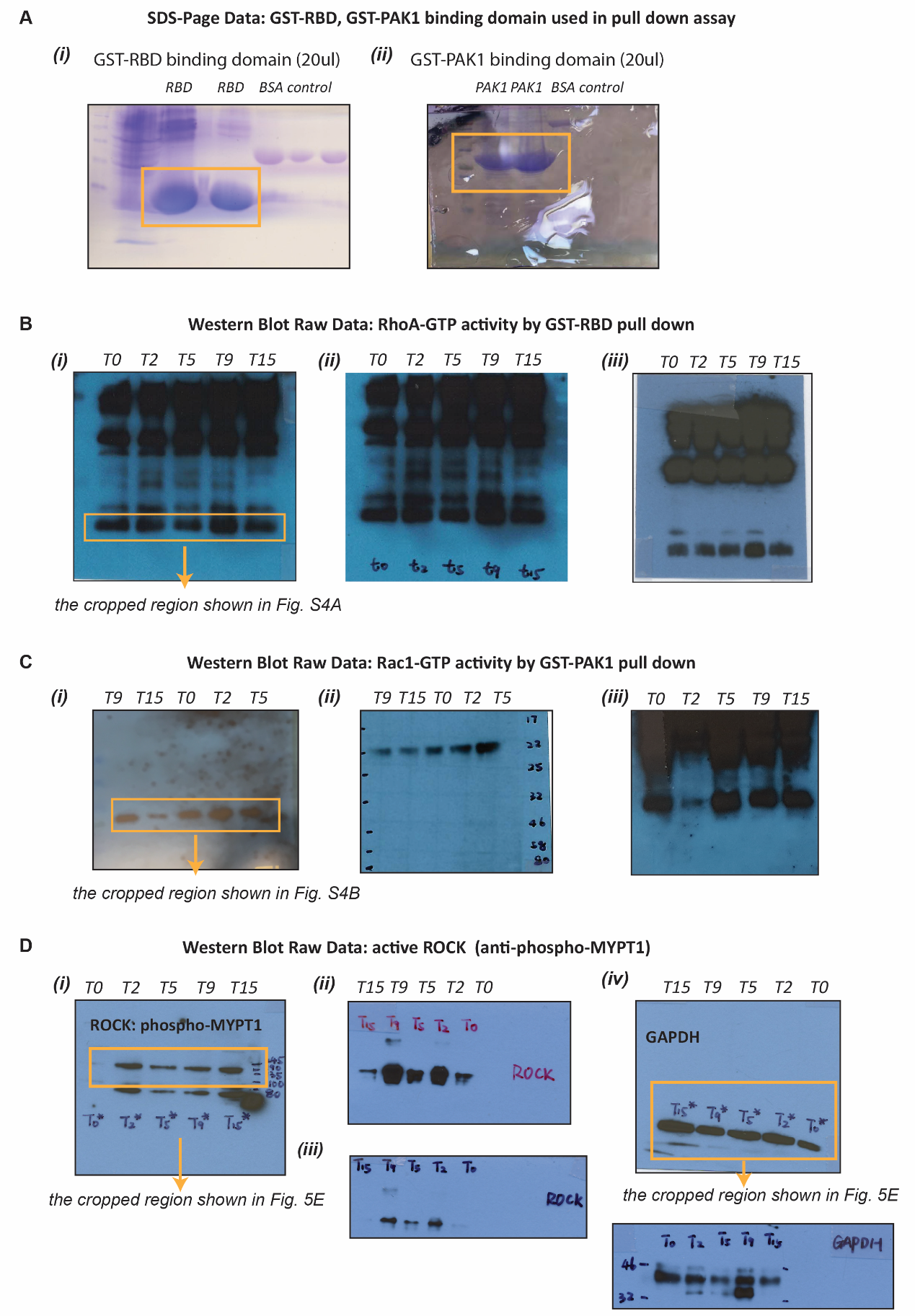
 Supplementary Figure 5. Original raw images related to Supplementary Fig. 4. (A) SDS-PAGE images of GST-RBD (left panel) and GST-PAK1 (right panel). These two recombinant protein domains were treated as the probes for fishing active RhoA and active Rac1, respectively. (B) Original images for blotting results of RhoA-GTP, which was pulled down by GST-RBD. Three different images were obtained from different experiments. The cropped part showed in Fig. S4A is highlighted. (C) Original images for blotting results of Rac1-GTP, which was pulled down by GST-PAK1. Three different images were obtained from different experiments. The cropped part showed in Fig. S4B is highlighted. (D) Western blotting images of phosphorylated myosin phosphatase subunit target 1 (phospho-MYPT1, Thr696), which represents ROCK activity, and housekeeping protein GAPDH. The images of phospho-MYPT1 and GAPDH were obtained from the same batch of cell extract.

**Supplementary Table 1. Statistical test results of Fig. 1F.**

| ***NIH 3T3 fibroblast: Speed*** | | | | | |
| --- | --- | --- | --- | --- | --- |
| *One-way-ANOVA-Result (statistic=48.03, p-value=8.39e-31)* | | | | | |
| *Multiple Comparison of Means - Tukey HSD, FWER=0.05* | | | | | |
| **group1** | **group2** | **mean diff** | **lower** | **upper** | **reject** |
| *E-G1* | *G2* | -0.1845 | -0.2516 | -0.1174 | True |
| *E-G1* | *L-G1* | -0.0882 | -0.1529 | -0.0234 | True |
| *E-G1* | *S* | -0.2798 | -0.3427 | -0.217 | True |
| *G2* | *L-G1* | 0.0964 | 0.0241 | 0.1686 | True |
| *G2* | *S* | -0.0953 | -0.1659 | -0.0248 | True |
| *L-G1* | *S* | -0.1917 | -0.26 | -0.1233 | True |
| ***NIH 3T3 fibroblast: Area*** | | | | | |
| *One-way-ANOVA-Result (statistic=472.8, p-value=2.0e-286)* | | | | | |
| *Multiple Comparison of Means - Tukey HSD, FWER=0.05* | | | | | |
| **group1** | **group2** | **mean diff** | **lower** | **upper** | **reject** |
| *E-G1* | *G2* | 1033.8488 | 931.5645 | 1136.1331 | True |
| *E-G1* | *L-G1* | 898.9655 | 800.2346 | 997.6964 | True |
| *E-G1* | *S* | 1113.4082 | 1017.6148 | 1209.2016 | True |
| *G2* | *L-G1* | -134.8833 | -245.0774 | -24.6893 | True |
| *G2* | *S* | 79.5594 | -28.0106 | 187.1294 | False |
| *L-G1* | *S* | 214.4427 | 110.2457 | 318.6398 | True |
| ***NIH 3T3 fibroblast: Aspect Ratio*** | | | | | |
| *One-way-ANOVA-Result (statistic=101.76, p-value=7.71e-65)* | | | | | |
| *Multiple Comparison of Means - Tukey HSD, FWER=0.05* | | | | | |
| **group1** | **group2** | **mean diff** | **lower** | **upper** | **reject** |
| *E-G1* | *G2* | -0.6367 | -0.7996 | -0.4738 | True |
| *E-G1* | *L-G1* | -0.0165 | -0.1738 | 0.1407 | False |
| *E-G1* | *S* | -0.9037 | -1.0563 | -0.7511 | True |
| *G2* | *L-G1* | 0.6202 | 0.4447 | 0.7957 | True |
| *G2* | *S* | -0.267 | -0.4383 | -0.0957 | True |
| *L-G1* | *S* | -0.8872 | -1.0532 | -0.7213 | True |
| ***NIH 3T3 fibroblast: Circularity*** | | | | | |
| *One-way-ANOVA-Result (statistic=44.3, p-value=2.02e-28)* | | | | | |
| *Multiple Comparison of Means - Tukey HSD, FWER=0.05* | | | | | |
| **group1** | **group2** | **mean diff** | **lower** | **upper** | **reject** |
| *E-G1* | *G2* | -0.0099 | -0.0234 | 0.0035 | False |
| *E-G1* | *L-G1* | -0.035 | -0.048 | -0.022 | True |
| *E-G1* | *S* | 0.0186 | 0.006 | 0.0312 | True |
| *G2* | *L-G1* | -0.025 | -0.0396 | -0.0105 | True |
| *G2* | *S* | 0.0285 | 0.0143 | 0.0427 | Ture |
| *L-G1* | *S* | 0.0535 | 0.0398 | 0.0673 | True |

**Supplementary Table 2. Statistical test results of Fig. 3E.**

| ***NIH 3T3 fibroblast: CMPI*** | | | | | |
| --- | --- | --- | --- | --- | --- |
| *One-way-ANOVA-Result (statistic=3.782, p-value=0.012)* | | | | | |
| *Multiple Comparison of Means - Tukey HSD, FWER=0.05* | | | | | |
| **group1** | **group2** | **mean diff** | **lower** | **upper** | **reject** |
| *E-G1* | *G2* | -0.1271 | -0.2432 | -0.011 | True |
| *E-G1* | *L-G1* | -0.0878 | -0.1998 | 0.0243 | False |
| *E-G1* | *S* | -0.1156 | -0.2243 | -0.0069 | True |
| *G2* | *L-G1* | 0.0393 | -0.0857 | 0.1644 | False |
| *G2* | *S* | 0.0115 | -0.1106 | 0.1336 | False |
| *L-G1* | *S* | -0.0278 | -0.1461 | 0.0904 | False |
| ***NIH 3T3 fibroblast: 3min-CCD*** | | | | | |
| *One-way-ANOVA-Result (statistic=48.03, p-value=8.39e-31)* | | | | | |
| *Multiple Comparison of Means - Tukey HSD, FWER=0.05* | | | | | |
| **group1** | **group2** | **mean diff** | **lower** | **upper** | **reject** |
| *E-G1* | *G2* | -0.465 | -0.5768 | -0.3531 | True |
| *E-G1* | *L-G1* | -0.3794 | -0.4874 | -0.2715 | True |
| *E-G1* | *S* | -0.7796 | -0.8844 | -0.6749 | True |
| *G2* | *L-G1* | 0.0855 | -0.035 | 0.206 | False |
| *G2* | *S* | -0.3147 | -0.4323 | -0.197 | True |
| *L-G1* | *S* | -0.4002 | -0.5141 | -0.2863 | True |

**Supplementary Table 3. Statistical test results of Fig. 4C.**

| ***Focal adhesions (FAs): Number*** | | | | | |
| --- | --- | --- | --- | --- | --- |
| *One-way-ANOVA-Result (statistic=3.82, p-value=0.025)* | | | | | |
| *Multiple Comparison of Means - Tukey HSD, FWER=0.05* | | | | | |
| **group1** | **group2** | **mean diff** | **lower** | **upper** | **reject** |
| *E-G1* | *G2* | -28.7037 | -77.5016 | 20.0942 | False |
| *E-G1* | *S* | -55.4259 | -103.3848 | -7.4671 | True |
| *G2* | *S* | -26.7222 | -80.0255 | 26.5811 | False |
| ***Focal adhesions (FAs): Area*** | | | | | |
| *One-way-ANOVA-Result (statistic=91.09, p-value=3.84e-40)* | | | | | |
| *Multiple Comparison of Means - Tukey HSD, FWER=0.05* | | | | | |
| **group1** | **group2** | **mean diff** | **lower** | **upper** | **reject** |
| *E-G1* | *G2* | 2.435 | 1.7183 | 3.1518 | True |
| *E-G1* | *S* | -1.5732 | -2.2357 | -0.9107 | True |
| *G2* | *S* | -4.0083 | -4.7047 | -3.3118 | Ture |
| ***Focal adhesions (FAs): Density*** | | | | | |
| *One-way-ANOVA-Result (statistic=1.92, p-value=0.151)* | | | | | |
| *Multiple Comparison of Means - Tukey HSD, FWER=0.05* | | | | | |
| **group1** | **group2** | **mean diff** | **lower** | **upper** | **reject** |
| *E-G1* | *G2* | -0.0069 | -0.021 | 0.0071 | False |
| *E-G1* | *S* | 0.0036 | -0.009 | 0.0163 | False |
| *G2* | *S* | 0.0106 | -0.0023 | 0.0234 | False |
| ***Stress Fibers (SFs): Number*** | | | | | |
| *One-way-ANOVA-Result (statistic=10.95, p-value=4.25e-05)* | | | | | |
| *Multiple Comparison of Means - Tukey HSD, FWER=0.05* | | | | | |
| **group1** | **group2** | **mean diff** | **lower** | **upper** | **reject** |
| *E-G1* | *G2* | -118.4368 | -198.3764 | -38.4973 | True |
| *E-G1* | *S* | -139.7685 | -218.3336 | -61.2034 | True |
| *G2* | *S* | -21.3317 | -108.652 | 65.9886 | False |
| ***Stress Fibers (SFs): Length*** | | | | | |
| *One-way-ANOVA-Result (statistic=66.52, p-value=1.44e-29)* | | | | | |
| *Multiple Comparison of Means - Tukey HSD, FWER=0.05* | | | | | |
| **group1** | **group2** | **mean diff** | **lower** | **upper** | **reject** |
| *E-G1* | *G2* | -2.0542 | -2.4536 | -1.6547 | True |
| *E-G1* | *S* | -0.4383 | -0.8266 | -0.0501 | True |
| *G2* | *S* | 1.6158 | 1.2189 | 2.0127 | Ture |
| ***Stress Fibers (SFs): Density*** | | | | | |
| *One-way-ANOVA-Result (statistic=0.84, p-value=0.43)* | | | | | |
| *Multiple Comparison of Means - Tukey HSD, FWER=0.05* | | | | | |
| **group1** | **group2** | **mean diff** | **lower** | **upper** | **reject** |
| *E-G1* | *G2* | 0.0089 | -0.0085 | 0.0263 | False |
| *E-G1* | *S* | 0.0065 | -0.0106 | 0.0236 | False |
| *G2* | *S* | -0.0024 | -0.0214 | 0.0166 | False |

**Supplementary Table 4. Statistical test results of Fig. 5B.**

| ***NIH 3T3 fibroblast: P27Kip1*** | | | | | |
| --- | --- | --- | --- | --- | --- |
| *One-way-ANOVA-Result (statistic=76.68, p-value=1.07e-47)* | | | | | |
| *Multiple Comparison of Means - Tukey HSD, FWER=0.05* | | | | | |
| **group1** | **group2** | **mean diff** | **lower** | **upper** | **reject** |
| *E-G1* | *G2* | -0.0569 | -0.0693 | -0.0445 | True |
| *E-G1* | *L-G1* | 0.0024 | -0.01 | 0.0148 | False |
| *E-G1* | *S* | 0.0139 | 0.0019 | 0.026 | True |
| *G2* | *L-G1* | 0.0593 | 0.0459 | 0.0727 | True |
| *G2* | *S* | 0.0708 | 0.0578 | 0.0839 | True |
| *L-G1* | *S* | -0.0115 | -0.0246 | 0.0015 | False |
| ***NIH 3T3 fibroblast: P27Kip1*** | | | | | |
| *One-way-ANOVA-Result (statistic=76.68, p-value=1.07e-47)* | | | | | |
| *Multiple Comparison of Means - Tukey HSD, FWER=0.05* | | | | | |
| **group1** | **group2** | **mean diff** | **lower** | **upper** | **reject** |
| *E-G1* | *G2* | -0.0569 | -0.0693 | -0.0445 | True |
| *E-G1* | *L-G1* | 0.0024 | -0.01 | 0.0148 | False |
| *E-G1* | *S* | 0.0139 | 0.0019 | 0.026 | True |
| *G2* | *L-G1* | 0.0593 | 0.0459 | 0.0727 | True |
| *G2* | *S* | 0.0708 | 0.0578 | 0.0839 | True |
| *L-G1* | *S* | -0.0115 | -0.0246 | 0.0015 | False |

**Supplementary Table 5. Statistical test results of Fig. 5D.**

| ***NIH 3T3 fibroblast: active-RhoA*** | | | | | |
| --- | --- | --- | --- | --- | --- |
| *One-way-ANOVA-Result (statistic=110.78, p-value=3.11e-70)* | | | | | |
| *Multiple Comparison of Means - Tukey HSD, FWER=0.05* | | | | | |
| **group1** | **group2** | **mean diff** | **lower** | **upper** | **reject** |
| *E-G1* | *G2* | 0.0367 | 0.0313 | 0.0421 | True |
| *E-G1* | *L-G1* | 0.0083 | 0.0025 | 0.0142 | True |
| *E-G1* | *S* | 0.0214 | 0.0154 | 0.0273 | True |
| *G2* | *L-G1* | -0.0284 | -0.0345 | -0.0223 | True |
| *G2* | *S* | -0.0153 | -0.0215 | -0.0091 | True |
| *L-G1* | *S* | -0.0131 | -0.0196 | -0.0065 | True |
| ***NIH 3T3 fibroblast: active-Rac1*** | | | | | |
| *One-way-ANOVA-Result (statistic=22.04, p-value=3.50e-14)* | | | | | |
| *Multiple Comparison of Means - Tukey HSD, FWER=0.05* | | | | | |
| **group1** | **group2** | **mean diff** | **lower** | **upper** | **reject** |
| *E-G1* | *G2* | -0.2057 | -0.2815 | -0.1299 | True |
| *E-G1* | *L-G1* | -0.0453 | -0.1222 | 0.0316 | False |
| *E-G1* | *S* | 0.0068 | -0.0672 | 0.0808 | False |
| *G2* | *L-G1* | 0.1604 | 0.0815 | 0.2392 | True |
| *G2* | *S* | 0.2125 | 0.1365 | 0.2886 | True |
| *L-G1* | *S* | -0.0521 | -0.1292 | 0.025 | False |

**Supplementary Table 6. Primary antibodies used in this study.**

| **Antibody Name** | **Company** | **Cat. #** | **Raised by** | **Mono. /Poly.** | **Dilution rate** | **Application** |
| --- | --- | --- | --- | --- | --- | --- |
| Vinculin | Sigma Aldrich | V9131 | Mouse | Mono. | 1:400 | IF |
| Active-RhoA-GTP | NewEast BioScience | 26904 | Mouse | Mono. | 1:100 | IF |
| Active-Rac1-GTP | NewEast BioScience | 26903 | Mouse | Mono. | 1:100 | IF |
| p21Cip1 (C-19) | Santa Cruz | Sc-397 | Rabbit | Poly. | 1:200 | IF |
| p27Kip1 (C-19) | Santa Cruz | Sc-528 | Rabbit | Poly. | 1:200 | IF |
| GAPDH | Santa Cruz | Sc-25778 | Rabbit | Poly. | 1:500 | WB |
| RhoA (26C4) | Santa Cruz | Sc-418 | Mouse | Mono. | 1:200 | WB |
| Rac1 (C-14) | Cytoskeleton Inc. | ARC03 | Mouse | Mono. | 1:200 | WB |
| Anti-phospho-MYPT1 (Thr696) | [EMD Millipore Corporation](javascript:openSupplierInfo(5697,'','',3,'SearchResults');) | ABS45 | Rabbit | Poly. | 1:1000 | WB |

*Mono.: Monoclonal; Poly.: Polyclonal; IF: Immunofluorescence; WB: Western blotting*
